# Translational Inhibition of CTX-M Extended Spectrum β-Lactamase in Clinical Strains of *Escherichia coli* by Synthetic Antisense Oligonucleotides Partially Restores Sensitivity to Cefotaxime

**DOI:** 10.3389/fmicb.2016.00373

**Published:** 2016-03-24

**Authors:** John B. Readman, George Dickson, Nick G. Coldham

**Affiliations:** ^1^Bacteriology Department, Animal and Plant Health AgencySurrey, UK; ^2^Centre for Biomedical Sciences, School of Biological Sciences, Royal Holloway, University of LondonSurrey, UK

**Keywords:** CTX-M, ESBL, translational inhibition, *E. coli*, antisense oligonucleotides, PMO, PNA

## Abstract

Synthetic antisense oligomers are DNA mimics that can specifically inhibit gene expression at the translational level by ribosomal steric hindrance. They bind to their mRNA targets by Watson-Crick base pairing and are resistant to degradation by both nucleases and proteases. A 25-mer phosphorodiamidate morpholino oligomer (PMO) and a 13-mer polyamide (peptide) nucleic acid (PNA) were designed to target mRNA (positions -4 to +21, and –17 to –5, respectively) close to the translational initiation site of the extended-spectrum β-lactamase resistance genes of CTX-M group 1. These antisense oligonucleotides were found to inhibit β-lactamase activity by up to 96% in a cell-free translation-transcription coupled system using an expression vector carrying a *bla*_CTX-M-15_ gene cloned from a clinical isolate. Despite evidence for up-regulation of CTX-M gene expression, they were both found to significantly restore sensitivity to cefotaxime (CTX) in *E. coli* AS19, an atypical cell wall permeable mutant, in a dose dependant manner (0-40 nM). The PMO and PNA were covalently bound to the cell penetrating peptide (CPP; (KFF)_3_K) and both significantly (*P* < 0.05) increased sensitivity to CTX in a dose dependent manner (0-40 nM) in field and clinical isolates harboring CTX-M group 1 β-lactamases. Antisense oligonucleotides targeted to the translational initiation site and Shine-Dalgarno region of *bla*_CTX-M-15_ inhibited gene expression, and when conjugated to a cell penetrating delivery vehicle, partially restored antibiotic sensitivity to both field and clinical isolates.

## Introduction

As a consequence of the well documented and ever increasing prevalence of antibiotic resistant bacteria, new methods of combating resistant strains which are pathogenic are urgently required for both animal and human health.

Particularly widespread in most countries in the northern hemisphere are the strains of *Escherichia coli* carrying group 1 *bla*_CTX-M_, conferring resistance to third generation cephalosporin antibiotics. First described in 1986, the CTX-M β-lactamases, named for their high level of activity against cefotaxime (CTX), and first observed in Munich (M), are a group of rapidly spreading ESBLs and are now the dominant family, especially found in such bacteria as *E. coli* and *K. pneumoniae* (Rossolini et al., 2008). They have spread globally and are common in many countries including the UK and USA (Canton et al., 2012). They appear to have a separate evolutionary history from other β-lactamases such as TEM and SHV and their ESBL-variants, with which they superficially share similar activity (Bonnet, 2003).

A variety of synthetic antisense/anti-gene agents, including phosphorodiamidate morpholino oligonucleotides (PMOs) and polyamide (peptide) nucleic acids (PNAs) have proven successful in inhibiting the expression of a diverse range of targeted bacterial genes (Rasmussen et al., 2007), demonstrating their potential to form the basis of novel antimicrobial therapeutic alternatives to traditional antibiotics (Woodford and Wareham, 2009; Goh et al., 2014). Synthetic translational inhibition agents have been shown to be effective *in vitro*, and in bacterial mutant strains with atypically permeable membranes, but have little activity in field isolates with intact cell walls, which restrict cytosolic entry (Good et al., 2000). However, when attached to a CPP, usually comprising of basic amino acids, the uptake of antisense agents in to the bacterial cell may be greatly increased (Shiraishi and Nielsen, 2011).

Phosphorodiamidate morpholino oligonucleotides are synthetic DNA analogs, incorporating natural DNA bases with a backbone consisting of a six membered morpholine ring with phosphorodiamidate linkages (Summerton et al., 1997). PNAs also comprised of natural DNA bases but a backbone of repeated *N*-(2-aminoethyl) glycine units linked by peptide bonds (Soomets et al., 1999). PNAs, with a lack of charge in the polyamide backbone, do not suffer from electrostatic repulsion associated with natural DNA-RNA (or combinations thereof) interactions (Armitage, 2003), which facilitates membrane transfer and yields a high DNA/RNA binding affinity. These oligonucleotides are resistant to enzymatic degradation, have a net neutral charge at physiological pH, and are able to block protein translation at the ribosome by the steric hindrance of complementary base pairing with mRNA (Lecosnier et al., 2011). The most effective area for translational protein expression inhibition in bacteria has been shown to be the Shine-Dalgarno (SD) ribosome binding site and the start codon region of the mRNA (Good and Nielsen, 1998; Dryselius et al., 2003).

Restoration of antibiotic sensitivity through translational inhibition of β-lactamase activity potentially might represent a viable therapeutic solution with the flexibility to quickly develop new inhibitory agents targeted to specific genes and perhaps restore the efficacy of certain now obsolete antibiotics. This is a potential alternative strategy at the translational molecular level for the treatment of CTX resistant bacteria. Resistance would be unlikely to evolve quickly in the highly conserved targeted ribosomal binding/translational start region.

Unmodified synthetic antisense oligonucleotides are known to be unable to efficiently penetrate the bacterial cell wall (Good et al., 2000). At around 2–3 kDa a 10-mer oligonucleotide is too large to enter a bacterial cell by porin-mediated passive transport (Nekhotiaeva et al., 2004), and therefore a delivery strategy to overcome this barrier is required. CPPs are short peptides of amino acids usually less than 30 residues in length, and usually consisting of a repeating pattern of positively charged and hydrophobic neutral residues, which are able to traverse a bacterial cell wall (Bechara and Sagan, 2013). A cargo molecule can be attached, usually by a direct covalent bond, to a CPP which is then translocated with the CPP across the cell membrane into the cytoplasm (Shiraishi and Nielsen, 2011). This approach has been shown to be effective in delivering synthetic antisense cargo molecules in eukaryotic cell lines (Shiraishi and Nielsen, 2011), and bacterial strains (Ghosal and Nielsen, 2012).

We have designed and developed two antisense oligo nucleotides, a 25-mer PMO (PMO1), and a 13-mer PNA (PNA4) to target areas of *bla*_CTX-M_ group 1 genes, inhibit β-lactamase expression and restore antibiotic sensitivity. We show sequence specific *bla*_CTX-M_ group 1 expression inhibition by a peptide conjugated PMO (P-PMO1) and a peptide conjugated PNA (P-PNA4), and a resultant phenotypic increase in sensitivity to the third generation cephalosporin antibiotic CTX in human clinical and veterinary field isolates.

## Materials and Methods

### Antisense Oligomers

PMO1 was synthesized by GeneTools, LLC (Philomath, OR, USA), with the base sequence of: agggtaccaattttttagtgacgcg (estimated purity > 92%). PNA4 was synthesized commercially by either Cellmano Biotech (Hefei, China) (purity D 95.51%), or Cambridge Research Biochemicals (Cleveland, UK) with the base sequence of: ggttattccttct. Peptide conjugated PMO1 (P-PMO1) and peptide conjugated PNA4 (P-PNA4) were synthesized commercially by Cambridge Research Biochemicals (Cleveland, UK), with the amino acid sequence of the CPP of KFFKFFKFFK.

### Strains

Strains used in this study are shown in **Table 1**. *E. coli* strains of human (clinical) origin were obtained from the Public Health laboratory, UK. AS19 was donated by Liam Good (RVC, London), BZ693/P, B3804 and LREC525 were from the collection of field strains at the Animal and Plant Health Agency. LREC90, used for control studies, carried a plasmid harboring *bla*_CTX-M-14_, which encoded β-lactamase from CTX-M-group 9. All other CTX resistant strains used in this study harbored variants of CTX-M-group 1.

**Table 1:** Details of strains used in this study.

Strain reference	Plasmid	*bla*_CTX-M_	Source Species	MIC–CTX mg/L	MIC–CTX + P-PMO1 (30 μM) mg/L	MIC–CTX + P-PNA4 (3.2 μM) mg/L
AS19 (Sekiguchi and Iida, 1967)	pJBRCTX516	CTX-M-15	Lab strain	80	4^†^	2^∗^
LREC460	pEK516 (Woodford et al., 2009)	CTX-M-15	Human	100	32^‡^	24
LREC454	pEK499 (Woodford et al., 2009)	CTX-M-15	Human	110	24	2
BZ693/P (Randall et al., 2011)	Not known	CTX-M-3/33	Chicken	40	15	12
B3804 (Freire Martin et al., 2014)	pIFM3804	CTX-M-1	Pig	48	10	2
LREC525 (Randall et al., 2011)	Not known	CTX-M-15	Turkey	260	96	48
LREC461	pEK204 (Woodford et al., 2009)	CTX-M-3	Human	35	16	8^•^
LREC90	Not known	CTX-M-14	Cattle	20	–	20


*References show either plasmid or strain characteristics/sequences. ^†^PMO, concentration of 40 μM; ^∗^PNA concentration of 20 μM; ^‡^P-PMO concentration of 40 μM; ^•^P-PNA concentration of 5 μM.*

### Construction of *bla*_CTX-M-15_ Expression Plasmid

Plasmid pJBRCTX516 was constructed carrying a single copy of *bla*_CTX-M-15_ cloned from a field isolate harboring plasmid pEK516 (Woodford et al., 2009). IS*Ecp*1 harbors at least one *bla*_CTX-M-15_ promoter (Poirel et al., 2003; Dhanji et al., 2011; Kamruzzaman et al., 2015). To ensure all native regulatory elements were included in the recombinant plasmid and that a valid genetic environment was replicated for an evaluation of the inhibitory potential of the antisense oligonucleotides, *bla*_CTX-M-15_ and the associated upstream insertional element IS*Ecp*1 was amplified by PCR (primers shown in **Table 2**, incorporating the restriction enzyme recognition sites *Xba*I and *Bam*HI). This amplicon was TA cloned into cloning vector pCR^TM^2.1-TOPO (Invitrogen). It was screened on LB agar supplemented with CTX (2 mg/L) and cultured in liquid LB overnight. The recombinant plasmid was digested with *Bam*HI and *Xba*I and the cloned insert isolated by gel purification. This was the ligated into the multi-cloning site (MCS) of the commercially available plasmid vector pET-9a (Novagen) and screened on LB agar supplemented with kanamycin (40 mg/L) and CTX (2 mg/L). The recombinant plasmid was designated pJBRCTX516. The successful construction of this plasmid was verified by restriction enzyme digestion and gel electrophoresis, and then by sequencing. An A to G point mutation at position +823 was corrected by site-directed mutagenesis using the GENEART^®^ Site-Directed Mutagenesis System (Invitrogen).

**Table 2:** PCR primer pairs for the amplification of *bla*_CTXM15_ and associated upstream region.

Designation	DNA Sequence	Amplicon	Amplicon
	(5′ to 3′)		Size (bp)
**CTXM-pEK516**			
Forward	ACTCA**agatct**GAAATTCAGCTTTCACCCATTG	CTX-M-15 and IS26 from pEK516	2552
Reverse	CCATGT**cctagg**CCGTTTCCGCTATTACAAAC		
**CTXM-pEK499**			
Forward	TACTGG**catatg**CGCTTGTGATTCGCCGCCGTCAGGTTGA	CTX-M-15 and IS*Ecp*1from pEK499	2119
Reverse	GAGCGT**ggatcc**CCGTTTCCGCTATTACAAAC		


*Restriction enzyme recognition sites are lower case bold.*

### Quantification of β-Lactamase CTX-M Activity

CTX-M enzyme activity was determined by measuring the degradation of CTX with an HPLC based assay. Overnight cultures of strains were prepared and grown in Mueller-Hinton Broth (MHB) to O.D._600 nm_ 0.6-0.8 (mid-log phase), with antibiotic supplements where required. Cell aggregates were disrupted by bead milling in a Retsch Mixer MM301 shaking homogeniser (F. Kurt Retsch GmbH, Haan, Germany) at full speed for 5 min with 0.2-0.4 mm glass beads. The cells were collected by centrifugation at 10,000*g* for 2 min and the supernatant retained. A suitable volume of supernatant was incubated at 37° C with the desired concentration of CTX solution for varying amounts of time. Enzymatic activity was halted by transferring incubation solution (125 μl) to pre-prepared HPLC vials containing 125 μl phosphate buffer (pH2.2). The HPLC method used was adapted from Nemutlu et al. (2009) for the detection of CTX and other cephalosporins. HPLC was performed using a Xterra C18 (250 mm × 4.6 mm, 5 μm, i.d.) column using Agilent Technologies 1100 series liquid chromatography system comprising of automated solvent delivery, sampler and array detector system. The following chromatographic conditions were used. Phosphate buffer (sodium dihydrogen orthophosphate dihydrate 40 mM; pH3.2) was mixed with 100% MeOH during automated analysis with a gradient pump to the following amounts (MeOH: Phosphate buffer); *t* = 0 mins 18:82%, *t* = 5 mins 18:82%; *t* = 15 mins 45:55%; *t* = 16 mins 55:45%; *t* = 21 mins 55:45%; *t* = 22 mins 18: 82%; *t* = 30 next injection at a flow rate of 0.85 ml/min, with an injection volume of 25 μl per sample. CTX was detected in HPLC effluent at a wavelength of 254 nm. Analysis was performed with ChemStation software (Agilent Technologies). CTX was quantified using a standard calibration curve (0-400 mg/L CTX). The activity of *bla*_CTX-M_ was expressed as CTX (μg) degraded per minute per bacterial cell.

### Inhibition of *bla*_CTX-M-15_ Activity by Synthetic Antisense Oligonucleotides in a Cell-Free Translation/Transcription Coupled System

The Expressway^TM^ Mini Cell-Free Expression System (Invitrogen^TM^) was used for all *in vitro* protein expression studies. The manufacturer’s supplied protocol was followed and the reaction mixture was incubated with plasmid pJBRCTX516 (1 ng), purified by peqGOLD Plasmid Miniprep Kit (Peqlab^®^ Ltd) and amount estimated by spectrophotometry. β-Lactamase activity was determined by HPLC as described above.

### Cell Growth Assay for Assessing the Activity of Antisense Oligonucleotides

The activity of antisense oligonucleotides was assessed by measuring their ability to restore the growth inhibitory effect of CTX in a cell growth assay. Bacterial cultures were typically grown from glycerol stocks at 37°C in a shaking incubator in MHB media containing CTX (2 mg/L) until early log phase (0.1–0.2 O.D._600 nm_) was achieved. The culture was diluted to achieve a final cell density of approximately 100,000 CFU/ml, previously established empirically to be optimal for assessing the effect of antisense oligonucleotides, and for consistency across experiments. Diluted cell suspension (50 μl) was incubated with MHB (50 μl) growth medium supplemented with antibiotics and inhibitory agents where required, and transferred to a 96 well microtiter plate (Falcon). CTX concentrations for *bla*_CTX-M_ inhibition studies were set at a level required to increase the dormant lag phase by 1-3 h, as established in the current study (not shown). This was determined to be an optimal concentration to yield observable effects of synergistic antisense-CTX activity and was strain dependent. Bacterial cell growth was measured in a BMG Labtech FluoStar automated spectrophotometer (BMG LABTECH GmbH, Ortenberg, Germany) and optical density readings (600 nm filter) taken over 18-24 h. The O.D._600 nm_ filter readings were taken over 250 cycles, with 15 flashes per well per cycle approximately every 5 min. All replicates were independent cultures. Growth curves and statistical analyses were produced using Prism^®^ 6 software (GraphPad). Minimum inhibitory concentrations (MICs) were determined to be the minimum antibiotic concentration observed by spectrophotometry required to completely inhibit growth in ≥50% of replicates over 18 h in the cell growth assay.

### Experimental Controls Used for the Evaluation of Inhibition of β-Lactamase Activity by Unmodified and Peptide-Conjugated PNA4/PMO1

**FIGURE 1:**
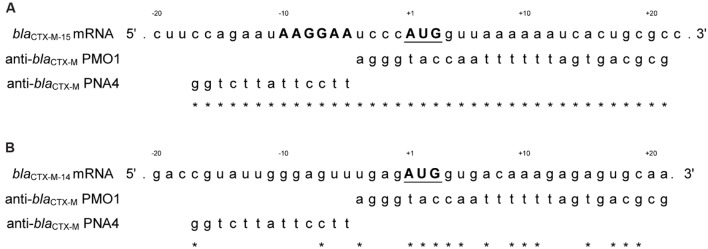
**(A)** Alignment of *bla*_CTX-M-15_ with PMO1 and PNA4. PMO1 was designed to target, by complementary base-pairing, to *bla*_CTX-M-15_ mRNA from positions -4 to +21. PNA4 was designed to target *bla*_CTX-M-15_ mRNA from positions -5 to +17. The Shine-Dalgarno region (SD) is marked in bold uppercase and the translational start codon region is bold uppercase and underscored. ^∗^represent complementary bases. **(B)** Lack of alignment from the start codon of *bla*_CTX-M-14_ with PMO1 and PNA4. *bla*_CTX-M-14_ is a mismatched control for anti-*bla*_CTX-M-15_ PNA4 and PMO1. Best alignment of PMO1 = 56% complementarity. Best alignment of PNA4 = 15% complementarity. ^∗^represent complementary bases.

Specificity and any inherent toxicity of peptide-conjugated and unmodified PNA4 and PMO1 were demonstrated in the following studies. A scrambled non-specific 25-mer PMO was incubated in the presence of pJBRCTX516 in a cell-free translation/transcription coupled system followed by the quantification of cefotaximase activity. AS19/pJBRCTX516 was cultured with the scrambled PMO in the presence and absence of CTX and growth measured by spectrophotometry over 18–24 h. Field isolates harboring *bla*_CTX-M-group1_ were cultured in the presence of P-PMO1 or P-PNA4 in the absence of CTX and growth measured by spectrophotometry over 18–24 h. The targeting of the non-essential *bla*_CTX-M_ gene allowed the use of P-PMO1 and P-PNA4 in the absence of CTX as a highly suitable control for isolating the effects of the antisense agents and/or CPP on growth. Controls in the absence of CTX were included with every study. Field isolate LREC90 harboring *bla*_CTX-M-14_ (non-complementary with anti-*bla*_CTX-M-15_ PNA4 and PMO1; **Figure 1B**) was cultured in the presence and absence of P-PNA4, P-PMO1, and CTX, and growth measured by spectrophotometry over 18–24 h. Minimum inhibitory concentrations (CTX) were established for LREC90 in the presence and absence of P-PNA4.

## Results

### Design of Antisense Oligonucleotides Targeting the Translation Initiation Region of *bla*_CTX-M-15_ mRNA

A 25 base PMO (PMO1) oligonucleotide was designed to target, by complementary base pairing, an area covering both the SD region and translational start codon region of *bla*_CTX-M-15_ mRNA (**Figure 1A**). However, due to potential hairpin self-dimerization and homo-dimerization, the design was modified under the advice of the manufacturer (Gene Tools LLC) and the final design was complementary to mRNA positions -4 to +21, with a sequence of: agggtaccaattttttagtgacgcg (**Figure 1A**). The target sequence was checked by NCBIs Basic Local Alignment Search Tool (BLAST) for homology with other bacterial and mammalian genes for potential unwanted off-target effects. This sequence was found by BLAST to be 100% complementary with all group 1 CTX-M β-lactamases. No bacterial off-target matches were found with greater than 72% sequence identity and no mammalian gene transcripts were found with a greater than 72% sequence identity.

A 13 base PNA (PNA4) was designed to target the SD region of *bla*_CTX-M-15_ mRNA (**Figure 1A**) with 100% sequence complementarity to the extragenic region -17 to -5. This target sequence was checked by BLAST and found to be to be 100% complementary with all group 1 CTX-M β-lactamases. Four bacterial off-target matches were found with 92.3% sequence similarity (12 out of 13 bases). A large number (>50) of off-target mammalian transcripts were detected (13 out of 13 bases).

### Construction of a Prokaryotic Expression Plasmid Harboring the *bla*_CTX-M-15_ Gene

**FIGURE 2:**
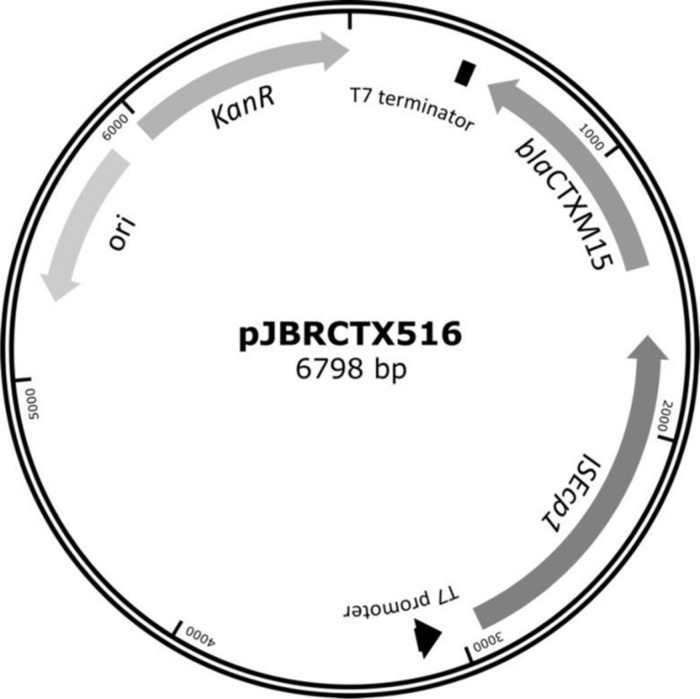
**β-Lactamase CTX-M-15 and its associated upstream insertional element IS*Ecp1* were cloned into the commercially available vector pET-9a to create an expression vector which, by virtue of the T7 promoter and termination sequences, could also be used in an *in vitro* cell-free transcription-translation coupled system.** This plasmid harbored no native β-lactamase genes, and kanamycin resistance for selection purposes.

Plasmid pJBRCTX516 was extracted and the successful recombination verified by restriction enzyme digestion and by sequencing (not shown). The plasmid vector contained T7 promoter and terminator regions flanking the MCS, making it a suitable vector for both cell-free and whole-cell inhibition experimental studies (**Figure 2**).

### Expression of *bla*_CTX-M-15_ in a Cell-Free Transcription/Translation System is Inhibited by Antisense Oligonucleotides

PMO1 significantly (*P* < 0.05) inhibited β-lactamase in a dose dependent manner (50-1000 nM). The following rates of CTX degradation were observed: uninhibited control sample: 1.01 μg/min/ng plasmid, 50 nM PMO: 0.34 μg/min/ng plasmid (66.05% inhibition), 100 nM PMO: 0.27 μg/min/ng plasmid (73% inhibition), 250 nM PMO: 0.15 μg/min/ng plasmid (85.33% inhibition), 500 nM PMO: 0.08 μg/min/ng plasmid (92% inhibition) and 1000 nM PMO: 0.04 μg/min/ng plasmid (96.08% inhibition). A non-complementary scrambled control PMO had no inhibitory effects on β-lactamase activity (not shown). PNA4, under the same conditions showed a similar, dose dependent effect ranging from 50% inhibition of CTX degradation at 50 nM PNA4, to 90% inhibition at 1000 nM PNA4.

### Levels of *bla*_CTX-M_ Activity in a Laboratory Cell-Wall Mutant Strain of *E. coli* are Reduced by Antisense Oligonucleotide Treatment

**FIGURE 3:**
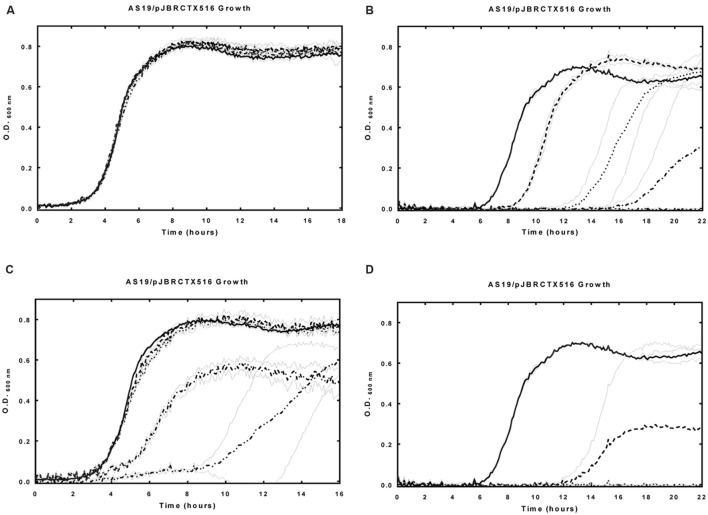
**Effects of unmodified anti-*bla*_CTX-M-15_ PMO1 and PNA4 alone, and in the presence of CTX on AS19/pJBRCTX516 growth.** *E. coli* strain AS19 harboring plasmid pJBRCTX516 was incubated in the presence and absence of PMO1, PNA4, and CTX with culture growth measured at 600 nm over 18–22 h. **(A)** Solid line: control, dashed lined: PMO1 (5 μM), dotted line: PMO1 (10 μM), dash–dot line: PMO1 (20 μM), dash–dot–dot line: PMO1 (30 μM). **(B)** Solid line: CTX (4 mg/L), dashed lined: CTX (4 mg/L) + PMO1 (10 μM), dotted line: CTX (4 mg/L) + PMO1 (20 μM), dash–dot line: CTX (4 mg/L) + PMO1 (30 μM), dash–dot–dot line (no growth): CTX (4 mg/L) + PMO1 (40 μM). **(C)** Solid line: control, dashed lined: PNA4 (5 μM), dotted line: PNA4 (10 μM), dash-dot line: PNA4 (20 μM), dash–dot–dot line: PNA4 (30 μM). **(D)** Solid line: CTX (4 mg/L), dashed lined: CTX (4 mg/L) + PNA4 (10 μM), dotted line: CTX (4 mg/L) + PNA4 (20 μM). Error bars illustrated in gray indicate ±1 SD (*n* = 2).

An increased sensitivity to CTX (4 mg/L) was shown in a dose dependant manner (20-40 μM PMO1 and 5-30 μM PNA4), with growth completely inhibited at PMO1 concentration of 40 μM (**Figure 3**). When treated with CTX (2 mg/L), 20 μM PNA4 was sufficient to completely inhibit growth (not shown).

There was no observed increase in CTX sensitivity when atypically permeable strain AS19/pJBRCTX516 was challenged with the combination of non-*bla*_CTX-M_ specific control PMO and CTX. In the absence of CTX, no effects on growth were observed when AS19/pJBRCTX516 was challenged with PMO1 alone (10–30 μM, **Figure 3**). In the absence of CTX, significant effects on growth were observed when AS19/pJBRCTX516 was challenged with PNA4 alone at concentrations higher than 5 μM (**Figure 3**). This was potentially attributable to unintended off-target partial complementarity with other bacterial genes. Consequently, PNA4 concentrations in subsequent studies did not exceed a concentration of 5 μM.

### Levels of *bla*_CTX-M_ Activity in Field and Clinical Isolate Strains of *E. coli* are Effectively Reduced by Treatment with Antisense Oligonucleotides Conjugated to a CPP

Field isolate LREC460 harboring the resistance plasmid pEK516 and field isolate LREC461 harboring the resistance plasmid pEK204, were incubated in Mueller Hinton Broth and challenged with CTX (16–32 mg/L) and PMO1/PNA4 (5–40 μM). It was found that neither PMO1 nor PNA4 had observable effects on growth or cell viability (data not shown). This was consistent with prior expectations as the cell wall has been previously shown to be a significant barrier to gene expression inhibition by antisense/antigene chemistries (Good et al., 2000).

A panel of clinical and field *E. coli* isolates were selected and MICs to CTX established (**Table 1**). These strains were challenged with a range of P-PMO1 and P-PNA4 concentrations in combination with the concentration of CTX required to extend the lag phase by 1-3 h (**Table 1**).

P-PMO1 was tested in combination with CTX in a range of six field and clinical isolates harboring plasmids carrying different group 1 CTX-M genes. All isolates tested showed an increased sensitivity to CTX when treated with anti-*bla*_CTX-M_ P-PMO1 (30 μM) ranging from a reduction in the MIC (to CTX) in *bla*_CTX-M-3_-harboring strain from 35 to 16 mg/L (2.19-fold increase in sensitivity), to a strain harboring *bla*_CTX-M-1_ from 48 to 10 mg/L (4.8-fold increase in sensitivity). The same isolates when treated with P-PNA4 (3.2 μM) had an observed increase in CTX sensitivity ranging from a reduction in MIC (to CTX) in *bla*_CTX-M-3/33_-harboring strain BZ693/P from 40 to 12 mg/L (3.3-fold) to a strain harboring *bla*_CTX-M-15_ (LREC454) from 110 to 2 mg/L (55-fold).

Any potential effects on bacterial culture growth not attributable to inhibition of *bla*_CTX-M-15_ were isolated by the treatment of cultures with P-PNA4 or P-PMO1 in the absence of CTX. In all strains tested, including strain LREC90 harboring *bla*_CTX-M-14_, both P-PMO1 and P-PNA4 in the absence of CTX individually had a small significant negative effect on growth, characterized by an extended lag phase period of variable, strain, and dose dependent amount. This was attributed to the effect of the CPP, which has been previously shown to have an impact on bacterial cell growth, and a synergistic effect with some antibiotics (Mohamed et al., 2014).

### Evaluation of the Sequence Specificity of Antisense Effects on Expression of CTX-M-15 Activity in Field Isolates

Previous studies have shown limited or no effect of scrambled or mismatch control peptide-conjugated PNAs on bacterial growth, including mismatch controls containing as few as two base substitutions (Ghosal and Nielsen, 2012). Studies with the peptide (KFF)_3_K attached to PNAs have demonstrated the efficacy of synthetic antigene oligonucleotides in bacteria, and their specificity by demonstrating low inhibition of target and reporter genes when challenged with scrambled non-specific peptide-conjugated PNAs (Good et al., 2001).

**FIGURE 4:**
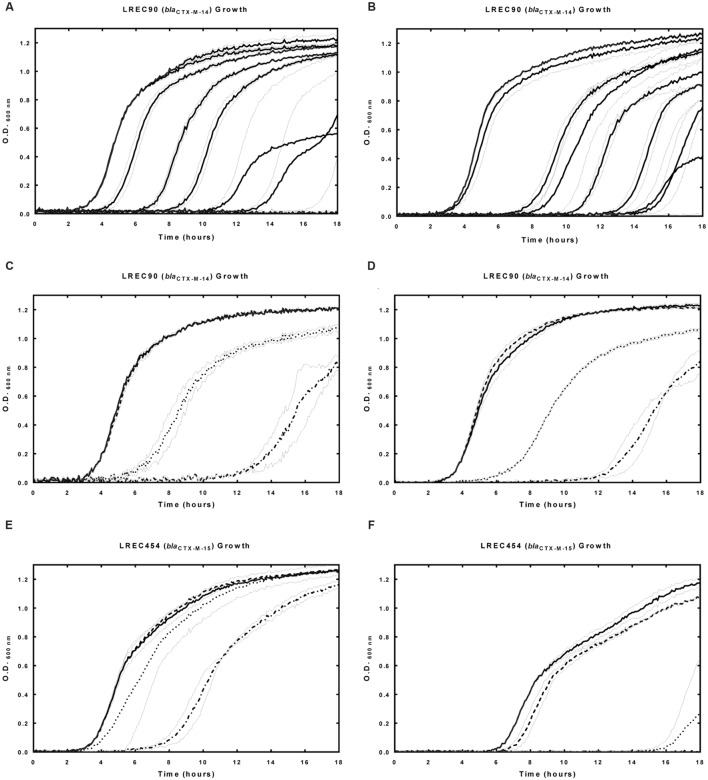
**Effects of P-PNA4 in the presence and absence of CTX on field isolate *E. coli* strain LREC90 (harboring *bla*_CTX-M-14_) and LREC454 (harboring *bla*_CTX-M-15_).** *bla*_CTX-M-14_ was a mismatched control for P-PNA4. Strains were incubated in the presence and absence of P-PNA4 and CTX with culture growth measured at 600 nm over 18 hours. **(A)** Field isolate LREC90 growth in the presence of increasing concentrations of CTX, lines from left to right: control, CTX (0 mg/L), CTX (2 mg/L), CTX (5 mg/L), CTX (8 mg/L), CTX (11 mg/L), CTX (14 mg/L), CTX (17 mg/L), CTX (20 mg/L). **(B)** Field isolate LREC90 in the presence of P-PNA4 (3.2 μM) and increasing concentrations of CTX, lines from left to right: P-PNA4 (3.2 μM), CTX (0 mg/L) + P-PNA4 (3.2 μM), CTX (2 mg/L) + P-PNA4 (3.2 μM), CTX (5 mg/L) + P-PNA4 (3.2 μM), CTX (8 mg/L) + P-PNA4 (3.2 μM), CTX (11 mg/L) + P-PNA4 (3.2 μM), CTX (14 mg/L) + P-PNA4 (3.2 μM), CTX (17 mg/L) + P-PNA4 (3.2 μM), CTX (20 mg/L) + P-PNA4 (3.2 μM). **(C)** Field isolate LREC90 in the presence of P-PNA4 (1.6–5 μM), solid line: control, dashed line: P-PNA4 (1.6 μM), dotted line: P-PNA4 (3.2 μM), dash–dot line: P-PNA4 (5 μM). **(D)** Field isolate LREC90 in the presence of CTX (2 mg/L) and P-PNA4 (1.6–5 μM), solid line: CTX (2 mg/L), dashed line: CTX (2 mg/L) + P-PNA4 (1.6 μM), dotted line: CTX (2 mg/L) + P-PNA4 (3.2 μM), dash–dot line: CTX (2 mg/L) + P-PNA4 (5 μM). **(E)** Field isolate LREC454 in the presence of P-PNA4 (1.6–5 μM), solid line: control, dashed line: P-PNA4 (1.6 μM), dotted line: P-PNA4 (3.2 μM), dash–dot line: P-PNA4 (5 μM). **(F)** Field isolate LREC454 in the presence of CTX (2 mg/L) and P-PNA4 (1.6–5 μM), solid line: CTX (2 mg/L), dashed line: CTX (2 mg/L) + P-PNA4 (1.6 μM), dotted line: CTX (2 mg/L) + P-PNA4 (3.2 μM), dash–dot line: CTX (2 mg/L) + P-PNA4 (5 μM). Error bars illustrated in gray indicate ±1 SD (*n* = 2).

Control studies, equivalent to the use of a scrambled non-specific P-PNA or P-PMO1, were undertaken in *E. coli* strain LREC90. PMO1 and PNA4 were non-specific to *bla*_CTX-M-14_ expressed by LREC90 (**Table 1B**). No significant differences were observed between the effects on growth of LREC90 cultured in the presence of P-PNA4 alone (1.6–5 μM), and P-PNA4 (1.6–5 μM) co-administered with CTX (2 mg/L). Minimum inhibitory concentrations (CTX) of LREC90 were not reduced in the presence of P-PNA4 (**Figure 4**). A comparable effect on LREC90 (*bla*_CTX-M-14_) and LREC454 (*bla*_CTX-M-15_) growth was observed when cultured in the presence of P-PMO1 (10–30 μM), attributable to the peptide portion of the conjugate.

**FIGURE 5:**
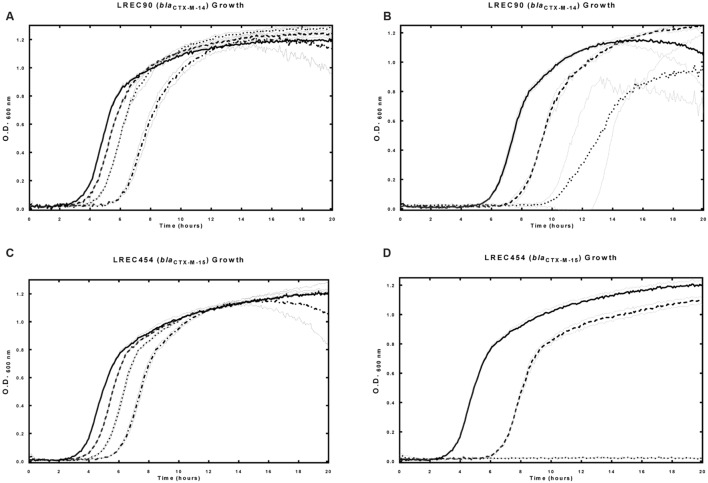
**Effects of P-PMO1 in the presence and absence of CTX on field isolate *E. coli* strain LREC90 (harboring *bla*_CTX-M-14_) and LREC454 (harboring *bla*_CTX-M-15_).** *bla*_CTX-M-14_ was a mismatched control for P-PMO1. Strains were incubated in the presence and absence of P-PMO1 and CTX with culture growth measured at 600 nm over 18 h. **(A)** Field isolate LREC90 in the presence of P-PMO1 (10–30 μM), solid line: control, dashed line: P-PMO1 (10 μM), dotted line: P-PMO1 (20 μM), dash–dot line: P-PMO1 (30 μM). **(B)** Field isolate LREC90 in the presence of CTX (3 mg/L) and P-PMO1 (10–30 μM), solid line: CTX (3 mg/L), dashed line: CTX (3 mg/L) + P-PMO1 (10 μM), dotted line: CTX (3 mg/L) + P-PMO1 (30 μM). **(C)** Field isolate LREC454 in the presence of P-PMO1 (10–30 μM), solid line: control, dashed line: P-PMO1 (10 μM), dotted line: P-PMO1 (20 μM), dash–dot line: P-PMO1 (30 μM). **(D)** Field isolate LREC454 in the presence of CTX (24 mg/L) and P-PMO1 (30 μM), solid line: control, dashed line: CTX (24 mg/L), dotted line: CTX (24 mg/L) + P-PMO1 (30 μM). Error bars illustrated in gray indicate ±1 SD (*n* = 2).

The negative effect on growth was increased in an additive manner (estimated fractional inhibitory concentration index (FICI) between 0.5 and 1) when LREC90 was cultured in the presence of P-PMO1 (10–30 μM) and CTX (3 mg/L), in contrast, the negative effect was increased in a synergistic manner (estimated FICI < 0.5) when LREC454 was cultured in the presence of P-PMO1 and CTX (24 mg/L) (**Figure 5**).

### Cephalosporin Treatment Induces Increased Levels of *bla*_CTX-M-15_ Activity in *E. coli* Strains

**FIGURE 6:**
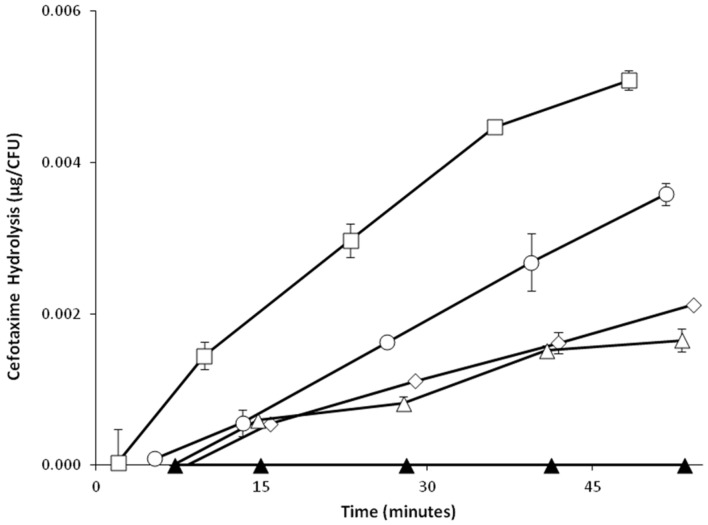
**Effect of exposure to the third generation cephalosporin ceftriaxone (CRO) on the upregulation of cefotaximase activity in recombinant *E. coli* strain AS19/pJBRCTX516.** *E. coli* cultures of strain AS19 harboring the plasmid pJBRCTX516, were incubated with concentrations of CRO (2-64 mg/L) for 30 min. Supernatants were collected, and incubated with CTX. A quantification of CTX degradation was established by HPLC in aliquots collected at regular intervals over 50 min. CRO concentrations were: zero (♢), 2 mg/L (△), 32 mg/L (○), and 64 mg/L (□). Filled triangles (▲) represent HPLC negative control blank sample. Error bars indicate **±**1 SD (*n* = 2).

The effect of exposure to an extended spectrum β-lactam on the activity of CTX-M-15 activity was investigated by pre-exposure to ceftriaxone (CRO; 2–384 mg/L) and subsequent quantification of CTX degradation. The rate of CTX degradation was increased in a dose dependent manner (2-64 mg/L) following exposure to higher levels of ceftriaxone in both AS19/pJBRCTX516 (**Figure 6**) and field isolate LREC460 (data not shown).

## Discussion

The application of antisense oligonucleotides for the translational inhibition of specific genes, such as those that confer resistance to ESBLs, is an attractive potential strategy for the restoration of antimicrobial sensitivity. PMOs and PNAs have been previously successfully employed to inhibit bacterial protein expression in a targeted, sequence specific manner. A wide range of bacterial essential and non-essential gene transcripts have been targeted and inhibited by synthetic antisense oligonucleotides. For example, a 17-mer PNA was reported by Lecosnier et al. (2011) to inhibit anti-insulin-like growth factor 1 receptor (IGF-1R) by 70–80% (Lecosnier et al., 2011). Geller et al. (2005) found that a 11-mer PMO significantly inhibited the viability *E. coli* through targeted inhibition of an essential gene (*acpP*) for phospholipid biosynthesis.

In this study, we have designed and evaluated a 25-mer PMO (PMO1) and a 13-mer PNA (PNA4) antisense/antigene oligonucleotide for efficacy of inhibition of translation of *bla*_CTX-M_ against a range of CTX-M group 1 β-lactamases in *E. coli.* This was demonstrated in a sequential series of progressive studies. β-lactamase was expressed in a cell free transcription/translation assay using a recombinant plasmid (pJBRCTX516) with a high amplitude promoter harboring *bla*_CTX-M-15_ as template DNA in the presence and absence of antisense oligonucleotides. β-lactamase activity was quantified by subsequent incubation with CTX, and degradation was quantified by an HPLC assay developed for the detection of CTX.

Increases in sensitivity to CTX in *E. coli* strains with reduced susceptibility to CTX (**Table 1**) were ascertained by cell growth assays in the presence of CTX and antisense oligonucleotides.

In cell free studies, PMO1 was found to inhibit production of *bla*_CTX-M-15_ in a dose-dependent (50-1000 nM) manner. At 1000 nM, β-lactamase production was inhibited by 96.08%. A scrambled control PMO of the same size, 25 nucleotides, non-specific to any bacterial gene, was found to have no inhibitory effects on transcription/ translation at this genetic level. These data suggest a high sequence specific inhibitory potential of PMO1 against *bla*_CTX-M-group1_. Under the same conditions, PNA4 was found to inhibit production of *bla*_CTX-M-15_ in a dose-dependent (50–1000 nM) manner with a maximum inhibition at 1000 nM of 89.39%.

PMO1 was tested against the atypically permeable cell-wall mutant AS19, transformed with pJBRCTX516 harboring *bla*_CTX-M-15_. Specificity was demonstrated at the phenotypic level as a scrambled control PMO with no specificity to bacterial genes had no observable effects on cell growth. There were also no observable effects when AS19/pJBRCTX516 was incubated with the maximum concentration used in inhibition studies (40 μM) PMO1 in the absence of CTX. There was a small observable lag phase extension effect, in a dose dependent manner (2.5-20 μM), when AS19/pJBRCTX516 was incubated with PNA4. This was attributed to the off-target matches associated with the sequence design of PNA4.

When incubated in the presence of both CTX and PMO1, increased antibiotic sensitivity was observed in a dose-dependent manner (10-40 nM). The MIC of CTX for this transformant, AS19/pJBRCTX516, was 80 mg/L, in the presence of 40 μM PMO1, this was reduced to 4 mg/L. These data demonstrated the lack of any inherent toxicity of PMO1 at the concentrations used, and the specific activity of PMO1 against *bla*_CTX-M-15_. Under the same conditions, incubated in growth medium additionally supplemented with PNA4 (20 μM), the minimum inhibitory concentration was reduced to 2 mg/L. At 20 μM, PNA4 alone was insufficient to inhibit growth and had a synergistic relationship with CTX (FICI < 0.5).

When tested with *E. coli* field isolates LREC460 and LREC461 containing plasmids harboring CTX-M group 1 genes, neither PMO1 nor PNA4 was found to have any effect on their sensitivity to CTX, or to their growth characteristics. This was not unexpected as previous studies have shown that *E. coli* with an intact cell wall do not efficiently take up naked antisense oligonucleotides (Good et al., 2000). To overcome this barrier, the anti-*bla*_CTX-M_ oligonucleotides PMO1 and PNA4 were covalently bound to the CPP, (KFF)_3_K, which has been shown to be effective in promoting uptake across the bacterial cell wall and delivering a cargo molecule to the cytoplasm (Good et al., 2001; Shiraishi and Nielsen, 2011). In the presence of peptide-conjugated PMO1 and PNA4, an increase in sensitivity to CTX was observed in all field isolates tested.

The disparity in the efficacy of P-PNA4 compared with P-PMO1 can, in part, be explained by the contribution of the off-target effects of the shorter PNA. Four bacterial gene transcripts shared 92.3% sequence identity with the *bla*_CTX-M_ target sequence (12 out of 13 bases), and an effect on growth was shown when challenging AS19/pJBRCTX516 with PNA4 alone. The off-target effects, however, were small in comparison with the synergistic effects of PNA4 in combination with CTX, and although significant (*P* < 0.05), were negligible at 3.2 μM. In the panel of six field and clinical isolates, P-PNA4 was found to be between 9- and 56-fold more effective in increasing sensitivity to CTX than P-PMO1.

When challenging the panel of strains with both P-PMO1 and P-PNA4 in the absence of CTX, a negative effect on bacterial cell growth was observed. This effect, not observed with unmodified oligonucleotides, can be attributed to the CPP portion of the conjugate. This effect has been noted by Bahnsen et al. (2013) and Mohamed et al. (2014) who also noted a strong synergistic effect of (KFF)_3_K with the antibiotics gentamicin and amikacin. It is likely that at low concentrations, the mechanism of cell penetration of (KFF)_3_K compromises the cell wall and increases the uptake of antibiotics, as has been observed in other bacterial species (Mohamed et al., 2014). Control studies using P-PMO1 and P-PNA4 in the presence and absence of CTX in strain LREC90 harboring *bla*_CTX-M-14_, to which anti-*bla*_CTX-M-15_ P-PMO1 and P-PNA4 had only partial sequence complementarity, revealed no synergistic relationship with CTX. In addition, when strains harboring *bla*_CTX-M-15_ were challenged with P-PMO1 and P-PNA4 alone, a comparable effect size to that shown in LREC90 was observed. Previously sub-lethal concentrations of CTX were sufficient to completely inhibit growth of these group 1 strains when in the presence of P-PMO1 or P-PNA4 at concentrations observed to have small or insignificant effects alone.

Specificity and the effect on growth of the CPP were demonstrated at the phenotypic level by the exposure of *E. coli* strain LREC90, harboring *bla*_CTX-M-14_ to CTX alone and in combination with P-PMO1 and P-PNA4. CTX-M-14, a group 9 CTX-M enzyme, shared 15.4% sequence complementarity (2 out of 13 bases) of the region targeted by P-PNA4, and 56% sequence complementarity (14 out of 25 bases) of the region targeted by P-PMO1. As such, this was regarded as a comparable control to scrambled non-specific antisense oligonucleotides in strains harboring *bla*_CTX-M-15._

A small effect on growth in the absence of CTX was observed, attributable to the CPP portion of the conjugate, potentially a result of membrane perturbation.

The relatively modest increase in sensitivity to CTX of field isolates when co-administered with P-PMO1, contrasted with a much higher level of re-sensitisation in the cell wall compromised strain AS19 transformed with pJBRCTX516, and a near complete inhibition of protein expression when tested in a cell-free environment. This disparity suggested either inefficient uptake of the antisense agent, a higher level of efflux in field isolates, or greater β-lactamase expression in response to CTX challenge.

We found experimental evidence to suggest that in conditions of increasing concentrations of CTX, in both field isolates and AS19/pJBRCTX516 there was a proportional up-regulation of β-lactamase activity. The differential expression mechanism requires further study.

The data obtained from translational inhibition studies of *bla*_CTX-M_ in field isolates, and the potential for further optimisation and enhancement, demonstrated the potential for therapeutic use of P-PMOs and P-PNAs in re-sensitizing resistant bacteria to CTX. To our knowledge this is the first study using synthetic oligonucleotides to demonstrate inhibition of *bla*_CTX-M_ group 1 genes in field and clinical isolates and restore sensitivity to the third generation cephalosporin CTX. Further studies are required to optimize uptake into the bacterial cell and translational inhibition.

## Author Contributions

JR: Ph.D. student, provided experimental work and wrote manauscript. GD: Academic PhD supervisor, provided guidance, and direction to experimental work and manuscript review/writing. NC: Industrial Ph.D. supervisor, provided guidance, and direction to experimental work and manuscript review/ writing.

## Conflict of Interest Statement

The authors declare that the research was conducted in the absence of any commercial or financial relationships that could be construed as a potential conflict of interest.
